# Biosafety Measures, Socio-Economic Impacts and Challenges of Bt-brinjal Cultivation in Bangladesh

**DOI:** 10.3389/fbioe.2020.00337

**Published:** 2020-05-14

**Authors:** Muhammad Shahidul Haque, Nihar Ranjan Saha

**Affiliations:** Department of Biotechnology, Bangladesh Agricultural University, Mymensingh, Bangladesh

**Keywords:** biosafety, Bt-crop, eggplant, farmers’ perception, fruit and shoot borer, pest management

## Abstract

This study surveyed the onsite biosafety measures adopted by the farmers cultivating Bt-brinjal, the socio-economic impact, and the challenges of Bt-brinjal cultivation in Bangladesh through interviews of 101 farmers from 26 Upazila (administrative region) under 20 Districts. Bt-brinjal 2, released by Bangladesh Agricultural Research Institute (BARI), is cultivated by 35% of the surveyed farmers. It was revealed that 52% of farmers maintained border crops. Among the growers, 52% informed that they disclose to the buyers that they are selling Bt-brinjal while selling in the open market where no product is traditionally labeled. Most of the farmers (71%) use Bt-brinjal plant debris as animal feed. Farmers (60%) received training on biosafety of Bt-brinjal cultivation. According to 85% of farmers, Bt-brinjal cultivation improved insect control. The farmers (77%) agreed that Bt-brinjal reduced labor and chemical costs and 75% of the farmers found increased yield and 72% of them found enhanced income by Bt-brinjal cultivation. However, 25% farmers informed that they did not get increased yield due to incidence of secondary insects. Most of the farmers (89%) perceive that cultivation of Bt-brinjal improved quality of brinjal. Furthermore, 59% of the farmers opined that price was reduced due to Bt-brinjal cultivation. The farmers also believe that Bt-brinjal cultivation reduced pesticide use (97%) and concern of insecticide use (96%) and hence they consider Bt-brinjal safer for human health (96%). However, to harvest the benefits of modern biotechnology, proper management of the biosafety in Bt-brinjal cultivation and labeling of Bt-brinjal during marketing should be maintained properly.

## Introduction

### Brinjal and Fruit and Shoot Borer (FSB)

Brinjal (*Solanum melongena L.*), also known as eggplant, is a popular multiuse vegetable cultivated in Asian countries, including Bangladesh. In Bangladesh, it is grown by about 150,000 farmers in 50,000 hectares of land, throughout the year in both the winter and summer seasons. The eggplant fruit and shoot borer (FSB) is responsible for the chronic and widespread infestation and considered the biggest constraint to eggplant production throughout Asia. FSB has become a major and regular pest of brinjal causing damage to even 30–50% of fruits or more in India, Bangladesh, Malaysia, Thailand, Burma, Sri Lanka, Laos, South Africa, Peoples Republic of the Congo. In severe cases, the infestation levels may exceed 90% and causing yield loss of up to 86% in Bangladesh (Ali et al., 1980). It affects the quality and quantity of fruits (Mall et al., 1992) rendering the fruits difficult to sell on the market and contains significantly less vitamin C (Abrol and Singh, 2003; Ghosh et al., 2003).

### Fruit and Shoot Borer (FSB) Control Measures

Farmers use tons of chemical pesticides annually to control pests that cause economic damage to crops. It was reported that 98% of the farmers rely solely on insecticide applications (Karim, 2004). The farmers spray insecticide almost every alternate day with as many as 84 applications in a cropping season (BARI, 1994). Not only in Bangladesh but also the Philippines, damage by FSB resulted in 80% yield loss of fruits and the control relies primarily on frequent applications of insecticides (Francisco, 2009). Consumers wish to avoid eating food that has been treated with pesticides because they are afraid of potential health hazards. The discharge of agricultural wastes from excessive use of pesticides and fertilizers can poison the water supply and cause harm to the environment. Moreover, pesticides are often applied without the appropriate protective equipment, resulting in high and prolonged exposures to farmers. Consequently, farmers suffer numerous health problems resulting from direct exposure to pesticide during handling and spraying (Rahman, 2000; Wilson and Tisdell, 2001). In Bangladesh, almost all farmers experienced sickness related to pesticide application, and 3% were hospitalized due to complications related to pesticide use (Alam et al., 2003). In India, 43% of the brinjal farmers suffered from health hazards due to various complexities related to pesticide application (Kolady and Lesser, 2005). Growing genetically modified (GM) Bt-crops (transgenic crops that produce the same toxin as the bacterium *Bacillus thuringiensis* in the plant cell, thereby, protecting the crops from pests) can reduce the application of chemical pesticides and the cost of bringing a crop to market (Moellenbeck et al., 2001).

### Release of Bt-brinjal and Cultivation

On 30 October 2013, Bangladesh approved the official release of four genetically modified, varieties of insect-resistant Bt-brinjal for seed production and initial commercialization. Bt-brinjal cultivation began in early 2014 in the spring season. The seedlings of four Bt-brinjal varieties were distributed to 20 small brinjal farmers on 22 January 2014. The farmers planted Bt-brinjal in a total area of 2.6 hectares in four representative regions of Gazipur, Jamalpur, Pabna, and Rangpur where these varieties are well-adapted and carefully monitored. Bt-brinjal-1 variety, popularly known as Uttara, was planted in Rajshahi region; Bt-brinjal-2 (Kajla) in Barisal region; Bt-brinjal-3 (Nayantara) in Rangpur and Dhaka regions; and Bt-brinjal-4 variety, Iswardi/ISD006, was planted in Pabna and Chittagong regions of the country. The Bangladesh Agricultural Development Corporation (BADC) in collaboration with BARI distributed seeds to farmers in the Kharif (Summer) season 2014. The government of Bangladesh planned to bring 20,000 hectares (40% of total 50,000 hectares) of land across 20 districts under Bt-brinjal cultivation. There are an estimated 150,000 brinjal farmers in Bangladesh, out of which 27,012 (∼17%) farmers are enjoying the benefits of the technology in 2018 (Shelton et al., 2018). Bt-brinjal is the first genetically modified (GM) Bt-food crop to be commercially cultivated in Bangladesh and in the world. Hence, the success of the Bt-brinjal cultivation, farmers’ profitability, the safety of environment and health and handling the future challenges efficiently can affect development and release of future genetically modified crops in Bangladesh, and other countries where biotechnology can play a vital role for food security and environmental safety.

### Biosafety in Bt-brinjal Cultivation

Handling transgenic crops in various stages require biosafety measures to ensure the conservation and sustainable use of biodiversity. Department of Environment in Bangladesh is responsible for ensuring biosafety measures through the implementation of Biosafety Rules and Guidelines (Biosafety Guidelines of Bangladesh, 2005; Anonymous, 2006b). The department helped the government to make decisions on genetically modified organisms (GMO) to be used in various conditions from lab to placement into the market. Although the government endorsed various uses of GMOs, there is no comprehensive information how the biosafety rules and guidelines are applied or followed by the farmers and benefits they are getting and also the challenges to be faced. Therefore, an initiative was taken to study the present status of the biosafety measures in post-release cultivation of Bt-brinjal to meet this information gap.

## Methodology

**FIGURE 1:**
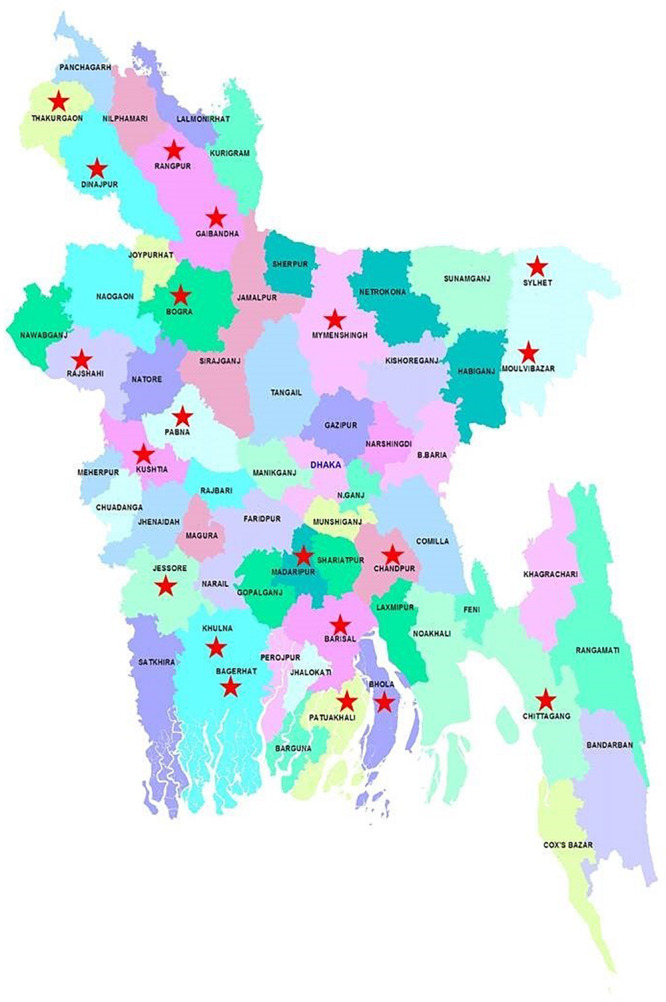
A map of Bangladesh showing the locations (*) where the survey was conducted. The map is a modification from that available in http://mapsof.net/uploads/thumbnails/500/bangladesh.png.

The study was conducted at 26 Upazila under 20 districts (Table 1 and Figure 1). The areas were randomly selected and were representative of all parts of Bangladesh. This study was conducted between March to May 2018 and consisted of interviews with 101 Bt-brinjal farmers. The surveyors visited Bt-brinjal cultivated field, talked to the farmers and consumers and collected data. An inclusion criterion was set for those farmers directly cultivating Bt-brinjal and focal farmer (farmers having regular contact with extension support staff), under the Department of Agriculture Extension (DAE). Farmers who had no land under DAE supervision were excluded from this survey.

**TABLE 1:** Name of the selected location to survey of Bt-brinjal cultivating area.

SI. No.	Name of district	Name of Upazila
1	Bogura	Gabtoli
2	Sylhet	Sylhet Sadar
		Gohainhat
		Bianibazar
3	Dinajpur	Dinajpur Sadar
4	Kushtia	Kushtia Sadar
5	Bager hat	Mollarhat
6	Moulovibazar	Juri
		Borolekha
7	Pabna	Pabna Sadar
8	Barishal	Babuganj
9	Khulna	Kotiaghata
10	Jessore	Jhikorgacha
11	Potuakhali	Dumki
12	Madaripur	Madaripur Sadar
13	Chittagong	Chittagong Sadar
14	Mymensingh	Gouripur
		Trishal
		Muktagacha
15	Tagurgao	Kaliadangi
16	Rangpur	Mithapukur
		Gongacora
17	Vola	Chorfashion
18	Rajshai	Puthia
19	Gaibandha	Polashbari
20	Chadpur	Kachua

We designed a questionnaire based on published literature (Anonymous, 2006a; Talukder, 2012) and the authors’ experiences in the field of biosafety. The questionnaire originally designed in English was translated into Bangla, the national language for the easy understanding of the farmers. Data were collected through a survey by face-to-face interviews with farmers and field observations during farming activities. The farmers were informed about the purpose of the study, and verbal consent was taken before the interview. The questionnaire consisted of four pages with 38 items. All items were rephrased as statements or a dichotomous statement (yes-no).

## Results

### Respondents’ Age, Educational and Socio-Economic Attributes

The study was conducted on 101 farmers who cultivate Bt-brinjal in 20 districts of Bangladesh. The farmers represented the mid-age group of 31–50 years followed by above 50, and below 30, respectively with diverse educational levels in the order of SSC (Secondary School Certificate) > write their name only > illiterate > above HSC (Higher Secondary Certificate). This study found that 67% of the Bt-brinjal farmers are subsistence farmers and they have less than 0.1 ha of land for Bt-brinjal cultivation. Only 5% of the Bt-brinjal farmers grew Bt-brinjal in 0.5–1.0 ha of land (Table 2). It shows that all the growers are marginal farmers having little or no profit from the farming but enjoying only a minimal livelihood. A recent report also found that nearly half of all brinjal farmers in both treatment and control groups are small farmers operating 0.5 to 1.49 acres of land (Ahmed et al., 2019). According to them, the second largest group is the medium farmer category, working 1.5 to 2.49 acres. The annual income of the farmers growing Bt-brinjal varied considerably. Most of the farmers belonged to the low-income group having their income below 20,000 Tk (Bangladeshi Taka) per annum. It reveals from Table 2 that 36% had an annual income below 10,000 Tk and 32% of the farmers had a yearly income below 20,000 Tk but above 10,000 Tk. However, 20% of the farmers had a higher income above 30,000 Tk per year.

**TABLE 2:** Basic information of the Bt-brinjal farmers.

Factor	Category	Percentage (%)
Age (years)	up to 30 years	15
	31–50	60
	> 50	25
Educational level	Illiterate	11
	Sign only	20
	SSC (Secondary School Certificate)	60
	HSC (Higher Secondary Certificate)	10
Farm size (ha)	Up to 0.1	67
	0.1 to 0.5	28
	0.5 to 1.0	5
Annul income	1,000–10,000 (low)	36
Bangladesh Taka (Tk)	11,000–20,000 (low)	32
	21,000–30,000 (medium)	12
	31,000–40,000 (high)	6
	> 41,000 (high)	14
Training on	Yes	60
Bt-brinjal cultivation	No	40

### Farmers’ Training for Bt-brinjal Growing

The study surveyed whether the farmers received any training that covered the biosafety measures to be taken for growing genetically modified Bt-brinjal and the process of cultivation of Bt-brinjal. Majority of the farmers (60%) under this study had exposure to training, while 40% had no training (Table 2). The farmers informed that BARI arranged 1 day training on Bt-brinjal cultivation for a limited number of farmers. It was reported that BARI, DAE and International Food Policy Research Institute (IFPRI) organized training of trainers, officers and farmers during 2017 covering various aspects of Bt-brinjal cultivation (Ahmed et al., 2019). Before the first release of Bt-brinjal in 2014, farmer training was conducted by BARI. More recently, the Department of Agricultural Extension (DAE) and the Agriculture Information Service (AIS) have become involved in training and distributing information on Bt-brinjal. However, the training was free of cost and did not cover all the farmers. The farmers without training hope that they should be given a minimum training on the safety and management of Bt-brinjal.

### Management Practices of Bt-brinjal Cultivation

#### Popularity of the Variety

Farmers of Bangladesh are cultivating four varieties of Bt-brinjal. Among the varieties, Bt-2 (Kazla) variety is grown by 35% of the targeted farmers, while 29% targeted farmers cultivate BARI Bt-brinjal 4 (ISD006). Only 7% of farmers are growing mixed; more than one varieties (Table 3). The Government of Bangladesh distributed seeds of four Bt-brinjal cultivars to different regions based on the history of consumers’ and farmers’ choice of the traditional counterpart of that GM brinjal cultivated. However, the farmers’ preference depends on the region of the country and the consumers’ choice over many years.

**TABLE 3:** Management practices of Bt-brinjal cultivation.

Factor	Category	Percentage (%)
Popularity of the varieties	Bt-brinjal −1	11
	Bt-brinjal −2	35
	Bt-brinjal −3	11
	Bt-brinjal −4	29
	Combine	7
Border crop management	Yes	52
	No	45
	No knowledge	3
Crop security management	Fencing	50
	Watchman	20
	No need	13
Pest management	Yes	58
	No	42
Harvesting the Bt-brinjal	Mix up Non-Bt-brinjal	62
	Non-mix up Non-Bt-brinjal	38
Labeling the Bt-brinjal for sale	Yes	52
	No	48
Debris management	Animal feed	71
	Burning	9
	Others	21

#### Management of Border Crops

**FIGURE 2:**
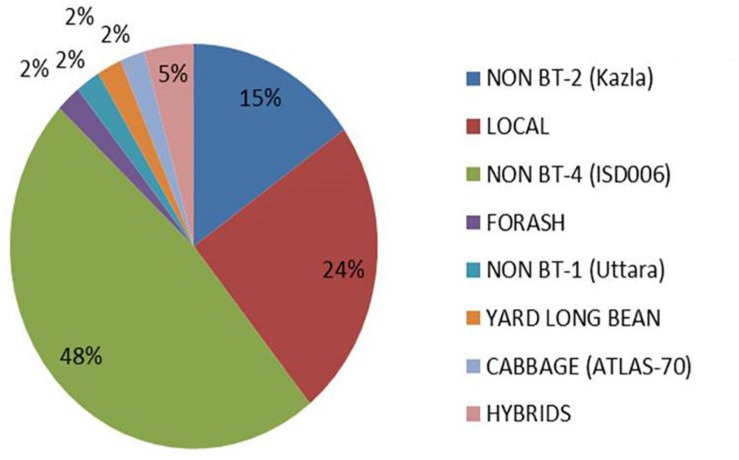
The border crops used around the Bt-brinjal fields.

The management of border crops is one of the most important safety aspects of GM crop cultivation. Cultivation of non-GM crop as a border crop around Bt-crop is advised for insect resistance management. In the case of Bt-brinjal, cultivation of 5% non-Bt-brinjal is necessary. It was found that more than half of the respondent farmers (52%) maintain border crop while growing Bt-brinjal in their fields. However, 45% of the farmers do not grow border crop, and only an insignificant percentage of farmers (<3% farmers) are unaware about the importance of growing border crops around the Bt-brinjal fields (Table 3). Although, more than 50% of farmers manage border crop, nearly equal percentage of farmers either do not manage or unaware of the matter. The farmers grow mostly non-Bt-brinjal as border crops. In 41% of the cases, farmers use non-Bt-brinjal variety ISD 006 while 24% of the farmers use local brinjal as border crop (Figure 2). It was found that 50% of the farmers used to fence around the Bt-brinjal field to protect the crop from animals. The others kept watching men for the protection of the crops (Table 3). BARI is continuing its effort in training focusing on the unique aspects of Bt-brinjal, mainly the requirements to plant a refuge of non-Bt-brinjal and the need to manage other “sucking insects” (Shelton et al., 2018). The survey indicated that 58% of farmers practised pest resistance management and applied insecticide to control insects other than FSB, while 42% did not apply any insecticide (Table 3). The data reveals that despite cultivating shoot and fruit borer resistant variety, 58% of the farmers are still afraid of other minor insects like mites and aphids. They want to keep their crop from minor insects. Therefore, they are using insecticides to protect brinjal from any loss.

#### Labeling of Bt-brinjal

The study results indicated that the majority (62%) of the farmers mix the Bt-brinjal with non-Bt-brinjals during harvesting (Table 3). In that case, the labeling cannot be done properly. Only 38% of the farmers are careful about separating the Bt-brinjal from non-Bt-brinjal. It indicates that the farmers are not very careful about the harvesting practice of the Bt-brinjal that is necessary for the labeling of Bt-brinjal.

In Bangladesh, labeling is rarely done for vegetables in the open market. It was found that 52% of Bt-brinjal growers inform the buyers that the brinjal is genetically modified while taking the product for sale in the market. In fact, they sold brinjal in open market where no labeling is traditionally practised in Bangladesh. The farmers informed that they just disclosed the buyers that the brinjal they are selling is Bt-brinjal. On the contrary, 48% growers did not mention the buyer that they are selling Bt-brinjal probably due to lack of training and knowledge or failure of understanding about the importance of the matter (Table 3).

#### Plant Debris Management

Management of the debris of the plants after harvest of Bt-brinjal is an important biosafety issue during containment and contained trials of GM crops. However, it is important to manage the debris of the plants after harvest of Bt-brinjal for biodiversity reason. Most of the farmers (71%) used the plant debris as animal feed, while 9% of farmers follow incineration or burning of the debris (Table 3). The rest 20% use them in various purposes like using as fuel in the kitchen or leave the debris in the field. The farmers are supplied with fresh seeds, and they normally do not keep the seeds for next year.

#### Benefits of Bt-brinjal Cultivation by Farmers

**FIGURE 3:**
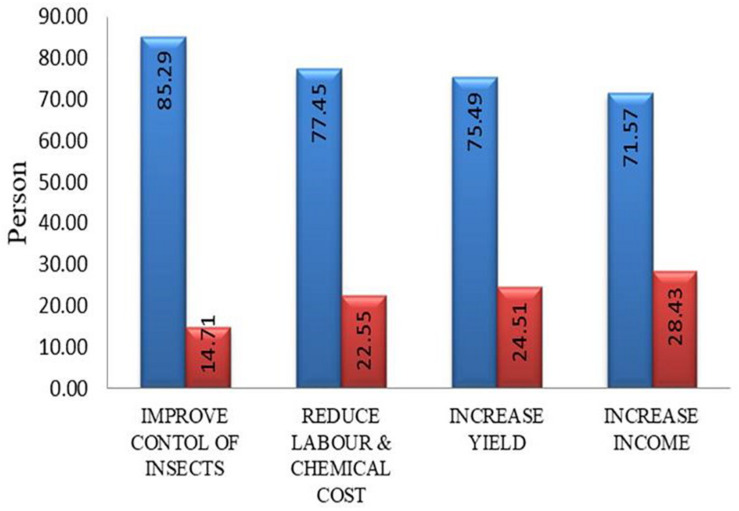
Benefits of Bt-brinjal cultivation by farmers.

Farmers benefit is the main component of GM brinjal cultivation as farmers are the primary stakeholder. Farmers will not cultivate Bt-brinjal if it is not profitable for them. In this survey, 85% of the farmers informed that cultivation of Bt-brinjal improved insect control (Figure 3). In comparison, 15% of the farmers disagreed with this opinion because there were other minor insects (e.g., whitefly) that caused damage to the Bt-brinjal, and Bt-brinjal had a higher number of leaves that caused hindrance to control insects. The basis of their disagreement was the presence of some minor insects in the field. Nearly 100% control of FSB by Bt-brinjal in the Philippines was reported (Hautea et al., 2016) with no negative impacts on non-target arthropods (Navasero et al., 2016). While in Bangladesh, as many as 6,500 farmers grew Bt-eggplant in 2017 and reaped its benefits (Shelton et al., 2017). Similar results were published from agronomic and socioeconomic studies conducted in Bangladesh (Shelton et al., 2018). Cultivation of Bt-brinjal reduced labor and chemical costs. Prodhan et al. (2018) compared the impacts of four Bt-brinjal varieties and conventional brinjal. They found a 0–2% fruit infestation of FSB among the Bt-brinjal varieties versus a 36–45% infestation in conventional brinjal varieties. Ahmed et al. (2019) reported FSB infested only 2% of all Bt-brinjal plants grown by the treatment farmers; by contrast, 34% of all ISD-006 brinjal plants grown by the control farmers were infested by FSB. These reports further demanded that Bt-brinjal has been successful in repelling FSB infestation and had no impact on non-target beneficial insects. A previous study by Rashid et al. (2018) assessed the impacts of four varieties of Bt-brinjal during the 2016/17 winter season and found that net returns were Tk 179,602 per ha for Bt-brinjal versus Tk 29,841 per ha for conventional brinjal (six times larger for Bt-brinjal farmers). Majority of the farmers (77%) agree with this opinion. In comparison, 23% told that there was no reduction of labor and chemical costs because they had to spray several times to control minor insects (Figure 3). These farmers reported that there was an incidence of secondary insects, and they had to spray insecticides to control those pests; thus, there was no considerable reduction in labor and chemical costs. Ahmed et al. (2019) claimed that overall, the cost of Bt-brinjal production per ha dropped by about 11% and cost per kg reduced by 31%. Again, 75% of the growers found increased yield due to growing Bt-brinjal while 25% of farmers informed that there was no increase in yield due to the incidence of secondary insects. A recent study reports that net yields were ∼40% higher for Bt-brinjal farmers compared to the conventional brinjal (Ahmed et al., 2019). The farmers were asked if they had an increase in income by Bt-brinjal cultivation; 72% growers replied that they were benefitted with an increase in income by Bt-brinjal cultivation. However, 28% of the growers replied that they did not find any increase in income because they did not get a good price of the brinjal (Figure 3). According to a recent report, the cost of production drops, mainly driven down by reduced pesticide costs, and revenues increase, mainly because of higher yields of Bt-brinjal and higher price. Increased production and a 10% reduction in costs, lead to a substantial increase in profits from cultivating Bt-brinjal that also conveys significant health benefits, both human and ecological while raising farmer incomes (Ahmed et al., 2019).

#### Farmers’ Perception of Bt-brinjal

**FIGURE 4:**
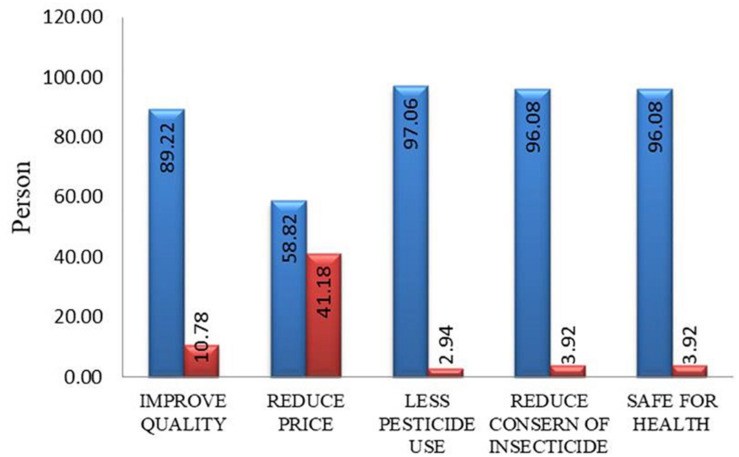
Farmers’ perception on Bt-brinjal.

Farmer satisfaction is a very important issue of Bt-brinjal cultivation as they are the most important stakeholder. If the farmers are not satisfied with Bt-brinjal, the cultivation is meaningless. This survey found that 89% of the farmers believe the cultivation of Bt-brinjal improved quality of brinjal, while 11% of farmers disagreed with this opinion (Figure 4). According to them, the taste was not the same as the non-Bt-brinjal. This disagreement might be the reflection of the differential performance of four varieties in various locations and individual choice of the farmers. Again, 59% of farmers opined that price was reduced due to Bt-brinjal cultivation because of higher production. In comparison, 41% did not find a reduction in price, so they believe that the adoption of Bt-brinjal cultivation did not reduce the price (Figure 4). The farmers (97%) informed that Bt-brinjal cultivation reduced pesticide use that they knew from the farmers and vegetable sellers and alleviated the concern of insecticide use (96%). Hence, they consider Bt-brinjal safe for human health (96%). However, 2–4% of the farmers did not agree with the above opinions.

**FIGURE 5:**
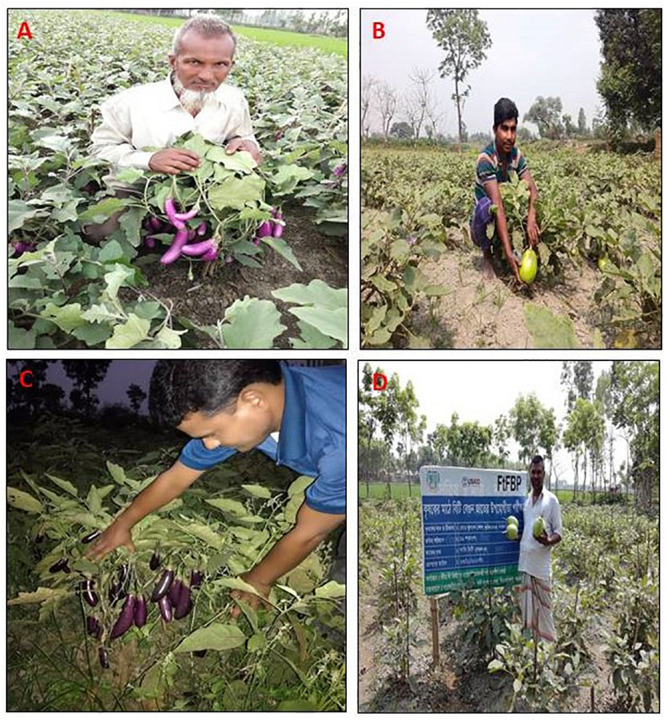
Farmers showing their harvest of Bt-brinjal cultivated in four different study locations of Bangladesh: **(A)** Kustia Sadar, **(B)** Dinajpur Sadar, **(C)** Rajshahi Puthia, and **(D)** Pabna Sadar.

A recent study found that Bt-brinjal released by Bangladesh government has cut toxicity of pesticides used by 41% and the farmers increased revenues by 27%. BARI scientists conducted a study in 35 districts during the 2016–2017 cropping season and reported that the farmers saved 61% of the pesticide cost compared to non-Bt-brinjal farmers and received higher net returns (unpublished, cited from Ahmed et al., 2019). Previous studies have shown that Bt-brinjal gave control of FSB and reduced insecticide use, with ultimate economic, health, and environmental benefits (Shelton et al., 2018). Because, it provides improved food safety, a more consistent supply of a highly nutritious vegetable, and less insecticide in the environment (Shelton et al., 2017). This study unveiled the fact that the farmers are happy with Bt-brinjal cultivation (Figure 5).

## Challenges Ahead

Every new approach faces challenges. Commercial cultivation of GM Bt-brinjal past few years has also generated concerns about its potential impacts on the environment, biodiversity and human and animal health. Ecological risk assessment of transgenic crops, issue of gene flow, development of secondary pest resistance and environmental risks involved with pollen flow are some of the issues related to any GM crop commercialization (Craig et al., 2008). People are confused about the risk that Bt-brinjal may pose to human health and the environment; the adequate follow up of guidelines, and the labeling for choice for consumers. Brinjal is historically a vegetable that is responsible for allergy to some people. Some media can use this information against Bt-brinjal as being allergic and toxic to both humans and animals. Currently, we did not find any strict practice of labeling to separate Bt-brinjal from non-Bt-brinjal. When both Bt-brinjal and non-Bt-brinjal are put on the market, people who would like to avoid GM food cannot exercise their right of choice.

An additional challenge associated with Bt-brinjal can result if there is a pest shift. A study in China showed that widespread adoption of Bt cotton and the associated decreased use of chemical insecticides have led to increased abundance of mirid bugs (Hemiptera: Miridae) in some fields (Lu et al., 2010). Another challenge in the sustainable use of Bt-technology is the evolution of resistance. As for other Bt-crops, over-reliance on Bt crops without appropriate Insect Resistance Management (IRM) or Integrated Pest Management (IPM) practices has led to a growing number of cases of target pest resistance (Gassmann et al., 2014; Tabashnik and Carriere, 2017). Legal court challenges against Bt-brinjal in India and the Philippines are another controversy. However, the court case filed against Bt-eggplant in the Philippines is more of a procedural issue than a technical one. An indefinite moratorium on Bt-brinjal for mass production in India is another challenge in Bangladesh. Once the court challenges against Bt-brinjal in India and the Philippines are solved, Bt-brinjal will quickly be popularized in Bangladesh.

The present study found very significant findings. The stakeholders, expressed their satisfaction with the performance of Bt-brinjal to a considerable level. The farmers reported that cultivation of Bt-brinjal improved insect control, reduced labor and chemical costs and increased yield and income. They are happy with quality brinjal at a lower price. Reduction in pesticide application and consequently, the reduced concern of insecticide use gave an impression to the farmers that Bt-brinjal is safe for human health. However, the study revealed a limited weakness in awareness, understanding and training among the farmers on Bt-brinjal cultivation and biosafety management and also labeling of GM product. Although different government agencies arranged the training on Bt-brinjal cultivation and biosafety management system, it was not sufficient.

Moreover, some farmers are reluctant to follow the instructions properly. Lack of supervision might be another cause behind the inadequacy of biosafety management by the farmers. In a country like Bangladesh, the marketing of vegetables lacks a proper labeling of the products, especially in the local village market. The lack of appropriate labeling system during the marketing of vegetables might have caused the absence of adequate labeling of Bt-brinjal during the wholesale and retail marketing of brinjal.

## Conclusion

Bt-brinjal is the first GM crop in Bangladesh. Some other GM crops are coming shortly. The success of Bt-brinjal cultivation can play an important role in the future of modern biotechnology in Bangladesh. The success in insect control, socioeconomic benefits to the farmers, and protection of environment, human and animal health of this first crop have set the stage for others to come. Fortunately, Bt-brinjal has a good start with increased yearly adoption and very favorable socioeconomic benefits. However, all farmers are not adequately aware of biosafety management practices and labelling of the GM brinjal is not done properly during selling them. Cultivation of Bt-brinjal facilitated control of insect, decreased insecticide use and increased yield. The reduction of pesticide application in Bt-brinjal gave farmers satisfaction. Monitoring and enforcement of the biosafety authority is also inadequate and needs need to be strengthened.

## Recommendation

Bt-brinjal is a genetically modified food crop. It is the first GM crop being cultivated in Bangladesh. The stakeholders are satisfied with the Bt-brinjal to a considerable level. The further development of modern biotechnology, development and cultivation of more GM crops to face the adverse effect climate change and the challenges to feed the increasing population of the country depend on the success of Bt-brinjal cultivation in the country. The survey revealed that labeling of the Bt-brinjal during placing into the market is not done properly which is needed to inform the consumer about the product as transgenic origin. To harvest the benefits of modern biotechnology, proper management of the biosafety and labeling of the product during marketing are highly recommended. Emphasis should be given on further training of the farmers, and supervision of the appropriate authority need to be strengthened towards ensuring management of pest resistance, border crop management practice and labeling of the product in the market. Further studies covering all the districts, farmers and consumers are recommended to establish a broader picture of the Biosafety measures adopted by the farmers on Bt-brinjal in Bangladesh.

## Data Availability Statement

All datasets generated for this study are included in the article/Supplementary Material.

## Ethics Statement

Written informed consent was obtained from the individual(s) and/or minor(s)’ legal guardian/next of kin for the publication of the identifiable images in this article.

## Author Contributions

Both the authors have contributions in the planning, surveying, and execution of the study. The authors contributed to the drafting and revision of the manuscript and subsequently approved the same for submission.

## Conflict of Interest

The authors declare that the research was conducted in the absence of any commercial or financial relationships that could be construed as a potential conflict of interest.
