# Supplementary material for: Biosafety Measures, Socio-Economic Impacts and Challenges of Bt-brinjal Cultivation in Bangladesh
Source: Front Bioeng Biotechnol. 2020 May 14;8:337. doi: 10.3389/fbioe.2020.00337 (PMC7247817; doi:10.3389/fbioe.2020.00337)
Supplement: Supplementary file 1 [file Table_1.pdf]

APPENDIX I
Department of Biotechnology
Bangladesh Agricultural University
Mymensingh
Survey on
“Bt-brinjal Cultivation”

Serial no (ক্রমিক নং)…………

Name of the respondent (নাম)……………………… Village (গ্রাম)……………………..….

Union (ইউনিয়ন) ……………….………… Upazilla (উপজেলা)……..…………….…………..

District (জেলা)………………………..…

Mobile No (মোবাইল নাম্বার): ………………………..………..….

Please furnish the information as mentioned in the following items (নিম্নলিখিত আইটেমগুলিতে উল্লিখিত তথ্য প্রদান করুন)

1. Age (বয়স): How old are you? ....Years. Gender (লিঙ্গ): Male/Female

2. Level of Education (শিক্ষার স্তর):

(Please mention your education level)
(আপনার শিক্ষা স্তর উল্লেখ করুন)

a) I can’t read and write ☐
b) I can sign only ☐
c) I studied…………………classes/passed………………examination

3. Farm size (জমির পরিমান): What‘s farm size according to use?

ব্যবহার অনুযায়ী থামারের আকার কি?

Land (জমি)	Land area (জমির পরিমান)	
	Local unit	Hectare (ha)
Land under Bt-brinjal Cultivation		

4. Annual income: Please tell me the amount of money (Tk) that you earned in last year?

বার্ষিক আয়: অনুগ্রহ করে বলুন যে আপনি গত অর্থবছরে যে পরিমাণ অর্থ উপার্জন করেছিলেন?

Sl. no.<br>ক্রমিক নং	Source of income (আয়ের উৎস)	Production<br>উৎপাদন	Price (taka)<br>মূল্য
1	Crops (ফসল)		
2	Bt-brinjal (বি. টি. বেগুন)		

5. Management Practices of Bt-brinjal Cultivation (বিটি বেগুন চাষের ব্যবস্থাপনা পদ্ধতি)

Please mention the duration you have been involved in Bt-brinjal Cultivation.

বিটি বেগুন চাষের সাথে জড়িত থাকার সময়কাল উল্লেখ করুন।

Years of involvement (জড়িত বছর)……………………………………….

I.

	Yes (হ্যাঁ)	No (না)	No knowledge<br>জ্ঞান নাই
Manage Border crop<br>সীমান্ত ফসল পরিচালনা করুন			

(a) If yes

Sl. no.<br>(ক্রমিক নং)	Crops Name<br>(ফসল নাম)	Variety Name<br>(ভ্যারাইটি নাম)	Height<br>(উচ্চতা)	Width<br>(প্রস্থ)
1				
2				

II. Name of the cultivated crops around the field (50-200m)

কাছাকাছি চাষযোগ্য ফসলের নাম (50-200 মি)

Sl. no. (ক্রমিক নং)	Crops Name (ফসল নাম)	Variety Name (ভ্যারাইটি নাম)
1		
2		
3		
4		

III. Any Pest Resistance Management

(কোন কীটপতঙ্গ প্রতিরোধ ব্যবস্থাপনা)

Yes (হ্যাঁ)	No (না)	No knowledge জ্ঞান নাই

(a) If yes

Sl. No. (ক্রমিক নং)	Brand Name (ব্রান্ড নাম)	Generic Name (জেনেরিক নাম)	Concentration (প্রয়োগ মাত্রা)	Result (ফলাফল)
1				
2				
3				

IV. Follow any instruction

কোন নির্দেশ অনুসরণ করেছেন?

Yes (হ্যাঁ)	No (না)

V. Harvesting Practice

ফসল কাটা পদ্ধতি

Mix up (মিশ্রিত)	Non Mix up (অমিশ্রিত)

VI. Label the Bt-brinjal for sale<br>বিক্রয় এর জন্য বিটি-বেগুন লেবেল করা হয়েছে কি না	Yes (হ্যাঁ)	No (না)	If No (cause) যদি না (কারণ)

VII. Is there any wild variety?<br>কোন দেশি জাত আছে?	Yes (হ্যাঁ)	No (না)

6. Training Exposure

Did you receive any training on Bt-brinjal Cultivation?

আপনি কি বিটি বেগুন চাষাবাদের প্রশিক্ষণ পেয়েছেন?

Yes (হ্যাঁ) ☐ No (না) ☐

If yes, please tell me the name and duration of training

যদি হ্যাঁ, তাহলে আমাকে প্রশিক্ষণ সম্পরকে তথ্য দিন

Sl. No. (ক্রমিক নং)	Name of the training প্রশিক্ষণের নাম	Duration of training (days) প্রশিক্ষণের সময়কাল (দিন)	Institution conducting training program প্রশিক্ষণ কোর্স পরিচালনা প্রতিষ্ঠান
1.			
2.			
3.			

7. Benefits of Bt-brinjal cultivation (Farmers / কৃষক)

Please mention your benefits with the Bt-brinjal cultivation

Improved control of insects<br>(কীটপতঙ্গ এর উন্নত নিয়ন্ত্রণ)	Yes (হ্যাঁ)	No (না)	If No (cause) যদি না (কারণ)

Reduced input costs such as labor and chemical (শ্রম ও রাসায়নিক হিসাবে কম খরচ)

Yes (হ্যাঁ)	No (না)	If No (cause) যদি না (কারণ)

Increased yields (ফলন বৃদ্ধি)

Yes (হ্যাঁ)	No (না)	If No (cause) যদি না (কারণ)

Increased yields (ফলন বৃদ্ধি)

Yes (হ্যাঁ)	No (না)	If No (cause) যদি না (কারণ)

Increased incomes (আয় বৃদ্ধি)

Yes (হ্যাঁ)	No (না)	If No (cause) যদি না (কারণ)

8. Benefits of Bt-brinjal cultivation (farmers / কৃষক)

Improved quality (মান উন্নয়ন)

Yes (হ্যাঁ)	No (না)	If No (cause) যদি না (কারণ)

Reduced price (মূল্য হ্রাস)

Yes (হ্যাঁ)	No (না)	If No (cause) যদি না (কারণ)

Less pesticide usage কম কীটনাশক ব্যবহার

Yes (হ্যাঁ)	No (না)	If No (cause) যদি না (কারণ)

Reduced concern of insecticide কীটনাশক হ্রাস করা উদ্বেগ	Yes (হ্যাঁ)	No (না)	If No (cause) যদি না (কারণ)

Safe for health স্বাস্থ্যের জন্য নিরাপদ	Yes (হ্যাঁ)	No (না)	If No (cause) যদি না (কারণ)

Thank you for your kind co-operation.

____

Signature of interviewer

Date:
